# Investigation of transcriptional and immunological disparities among patient groups with varied prognostic risk factors in cholangiocarcinoma

**DOI:** 10.1002/cam4.70135

**Published:** 2024-08-29

**Authors:** Baoluhe Zhang, Bao Jin, Xiang'an Wu, Jiali Xing, Xiao Liu, Xueshuai Wan, Haifeng Xu, Yiyao Xu, Yilei Mao, Qian Chen, Yating Bai, Mei Guan, Shunda Du

**Affiliations:** ^1^ Department of Liver Surgery Peking Union Medical College Hospital, Chinese Academy of Medical Sciences and Peking Union Medical College Beijing China; ^2^ Thorgene Co., Ltd. Beijing China; ^3^ Department of Medical Oncology Peking Union Medical College Hospital, Chinese Academy of Medical Sciences and Peking Union Medical college Beijing China

**Keywords:** cholangiocarcinoma, differentiation, immune, integrated multiomics analysis, lymphatic node transfer

## Abstract

**Background:**

This study explores molecular features associated with better prognosis in cholangiocarcinoma (CCA).

**Methods and Results:**

The transcriptomic and whole‐exome sequencing data obtained from paired tissues of 70 were analyzed, grouping them based on progression‐free survival (PFS), differentiation degree, and lymph node metastasis. Among the 70 patients, the TP53 gene mutation frequency was the highest (53%), while FLG gene mutation occurred exclusively in the long PFS group. In the comparison between long and short survival groups, the short PFS group exhibited higher monocyte infiltration levels (*p* = 0.0287) and upregulation of genes associated with cancer‐related transcriptional misregulation, chemokine signaling, and cytokine‐cytokine receptor interactions. Differences in immune cell infiltration and gene expression were significant across differentiation and lymph node metastasis groups. Particularly noteworthy was the marked increase in CD8 T cell and NK cell infiltration (*p* = 0.0291, 0.0459) in the lymph node metastasis group, significantly influences prognosis. Additionally, genes related to platinum resistance, Th17 cell differentiation, and Th1 and Th2 cell differentiation pathways were overexpressed in this group. In summary, higher monocyte infiltration levels in the short PFS group, along with elevated expression of genes associated with cancer‐related pathways, suggest a poorer prognosis. The significant increase in CD8 T cell and NK cell infiltration reflects an enhanced anti‐tumor immune response, underscoring the relevance of immune infiltration levels and gene expression in predicting outcomes for CCA patients.

**Conclusions:**

In this study, we elucidated the pertinent molecular mechanisms and pathways that influence the prognosis of CCAs through comprehensive multi‐omics analysis.

## 1 INTRODUCTION

Cholangiocarcinoma (CCA), originating from biliary duct cells, is a highly lethal malignancy.^1^, ^2^ Despite its lower incidence compared to other malignancies, global CCA rates are rising.^3^, ^4^, ^5^, ^6^ Peak onset is typically at 60–70 years, more common in men.^7^ Most CCAs are diagnosed at advanced stages, with only 30% being curable,^8^ resulting in a poor prognosis (5‐year survival: 7%–20%).^4^, ^9^ According to previous studies, genetic abnormalities,^10^ immune infiltration,^11^ tumor stage,^12^ and lymph node metastasis^10^ are generally considered as predictors of tumor prognosis. However, exploring new prognostic biomarkers, especially for different differentiations, is crucial. With advancements in next‐generation sequencing (NGS), analyzing immune cell infiltration through transcriptomic data is feasible. Immune cells play complex roles in anti‐tumor processes, influencing prognosis by activating cytotoxic T cells or expressing immunosuppressive factors.^13^ The efficacy of immunotherapy in some cancers, treated with immune checkpoint inhibitors (ICI),^14^, ^15^ correlates strongly with tumor mutational burden (TMB) and clinical benefit.

This study aimed to identify molecular features associated with a better prognosis in CCA patients, seeking potential biomarkers for prognosis prediction and treatment optimization. Tissues from 70 CCA patients were collected for whole exome sequencing (WES) and RNA sequencing. Groups were stratified based on tumor differentiation, lymph node metastasis, and progression‐free survival (PFS) length for a comprehensive analysis of molecular features, including somatic mutations, TMB, intra‐tumor heterogeneity (ITH), immune infiltration, and differentially expressed genes (DEGs).

## 2 MATERIALS AND METHODS

### 2.1 Patients and sample characteristics

Tumor and blood specimens from 70 CCA patients at Peking Union Medical College Hospital (2018–2022) were collected. Inclusion criteria were: (a) histologically confirmed CCAs, (b) age 18–82, (c) no gender restriction, (d) sufficient tissue for sequencing, (e) no severe comorbidities, and (f) regular postoperative follow‐up. All cases were verified by two independent liver pathologists. The results showed that among the 70 patients with CCA, 58 had distal cholangiocarcinoma, 9 had intrahepatic cholangiocarcinoma, and 4 had perihilar cholangiocarcinoma. Due to the limited number of intrahepatic and perihilar cholangiocarcinoma samples, further subtype differentiation was not performed. Additionally, none of the samples had received immunotherapy. The study, approved by Ethics Review Committee of Peking Union Medical College Hospital (I‐23PJ1691), aimed to analyze gene mutations, expression, and immune infiltration. The 70 CCA samples were categorized into two groups based on tumor differentiation (Group1: moderately to highly differentiated; Group2: poorly differentiated). Samples were further classified into groups with and without lymph node metastasis. Additionally, 37 samples with PFS data were stratified into long‐term (PFS ≥38.1 weeks) and short‐term (PFS <38.1 weeks) groups according to the median (Table S1 for grouping details).

### 2.2 WES and somatic mutation analysis

DNA samples from tumor and normal formalin‐fixed paraffin‐embedding (FFPE) tissues were obtained using the QIAamp DNA FFPE Tissue Kit (Qiagen). For fresh tumor and matched blood samples, the TIANamp Genomic DNA Kit (Tiangen Biotech) was used. DNA libraries were created with the SureSelect Kit (Agilent), and WES was conducted on the HiSeq X10 platform (Illumina Inc.). Library construction involved DNA fragmentation, end repair, adenylation at 3′ ends, end connection, amplification, purification, and size selection. Sequencing was performed on the Illumina X10 platform, and mutation analysis of WES data utilized GATK MuTect2. Reads were realigned to hg19 using Burrows‐Wheeler Aligner BWA‐MEM for improved single‐nucleotide variation (SNV) validity.

### 2.3 RNA‐seq and gene expression

Tumor RNA was extracted from FFPE tissues using the RNeasy FFPE kit (Qiagen). Libraries for NGS sequencing were constructed with the TruSeq RNA Exome kit. For fresh tissue, tumor RNA was extracted using TRIzol® reagent (Invitrogen). NGS sequencing for fresh tissue libraries utilized the NEBNext® UltraTM II RNA Library Prep Kit (NEB). Total RNA underwent fragmentation, reverse transcription, addition of ‘A' base at the 3’ end, ligation to adapters, amplification, purification, and sequencing on the Illumina X10 platform. Clean reads were mapped to the human hg19 genome using Bowtie2 and Tophat2 with default parameters. Gene expression levels, measured in Fragments Per Kilobase of transcript per Million mapped reads (FPKM), were calculated using the Cufflinks program.

### 2.4 Identification of differentially expressed genes and Kyoto Encyclopedia of Genes and Genomes pathway enrichment

DEGs were identified using Cuffdiff (version 2.2.1, default parameters). Significant DEGs met the criteria of absolute log2 (fold change) ≥1 and *p* < 0.05. Kyoto Encyclopedia of Genes and Genomes (KEGG) function enrichment analysis was conducted with KEGG Orthology Based Annotation System (KOBAS) (version 2.1.1, default parameters). Enriched pathways with *p* < 0.05 were considered statistically significant.

### 2.5 Gene set enrichment analysis

Gene set enrichment analysis (GSEA) (version 4.0.3, default parameters) identified significant pathways using the KEGG subset of CP in C2 curated gene sets from MSigDB (http://software.broadinstitute.org/gsea/msigdb) as the reference gene sets. Significance thresholds were determined through permutation analysis (1000 permutations). Results with |NES| >1, nominal *p*‐value <0.05, and false discovery rate *q*‐value <0.25 were considered statistically significant.

### 2.6 Immune cell infiltration and ICI marker expression

Immune infiltration was estimated using ESTIMATE software (version 1.0.13, default parameters) to calculate the fraction of stromal and immune cells in tumor samples based on FPKM, characterizing immune cell infiltration with the ‘Immune score.’ Absolute cell abundance in the tumor was estimated using microenvironment cell populations (MCP)‐counter software (version 1.1.0, default parameters), encompassing eight immune cells and two stromal cells. The ESTIMATE algorithm was further utilized to calculate the Immune Score and CYT_score, and immunotherapy effectiveness was predicted by comparing the expression levels of ICI marker genes.

### 2.7 Validation of ICI marker by immunohistochemical assay

Selected ICI, including PD1, PDL2, CTLA4, IDO1, TIGIT, LAG3, and VSIR, were used for immunohistochemical staining. Antibodies were obtained from Abcam (USA) and Bailing Bio (China). The main experimental steps included the following deparaffinization of paraffin sections, antigen retrieval using various solutions, blocking of endogenous peroxidase for 15 min, washing with PBS buffer for 5 min, blocking with goat serum for 1 h at room temperature, incubation with the primary antibody, washing with PBS buffer for 5 min (repeated three times), incubation with enzyme‐labeled goat/rabbit IgG polymers (ZSGB‐BIO, Universal Kit, PV‐6000) for 30 min at room temperature, DAB color development, hematoxylin re‐staining, dehydration, clearing, and sealing with neutral gum.

### 2.8 Statistical analysis

Statistical analysis was performed using GraphPad Prism 8.0 software (GraphPad Software, San Diego, USA). Group comparisons involved two‐sided Fisher's exact test for categorical variables and two‐tailed unpaired *t*‐test for numerical variables. Survival differences were calculated using the log‐rank test. A significance level of *p* < 0.05 was applied. Figures were generated using GraphPad Prism 8.0 software or R (version 4.2.1, https://cran.r‐project.org/).

## 3 RESULTS

### 3.1 Patient characteristics and somatic and gene mutation profiles

**FIGURE 1:**
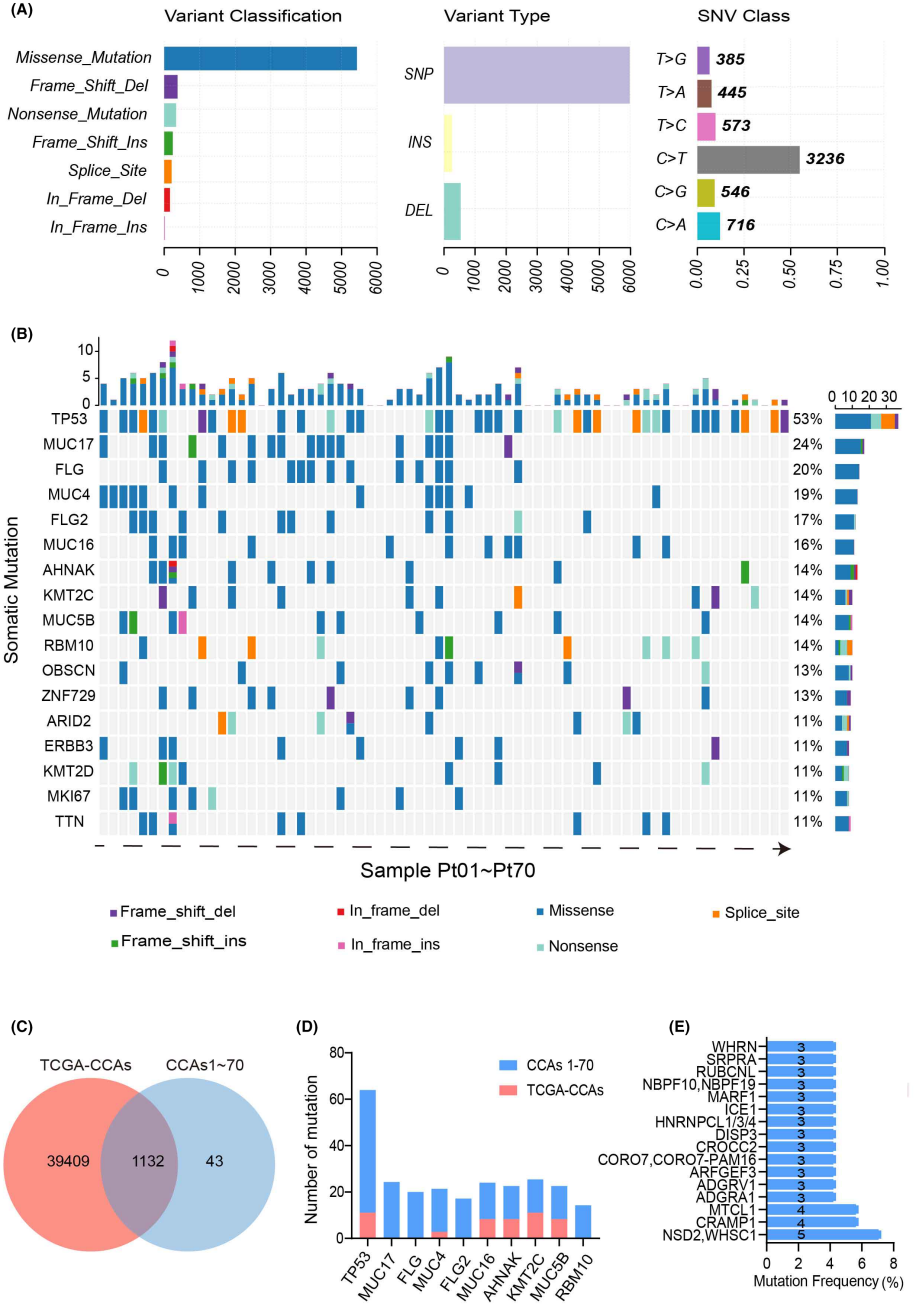
Somatic cell mapping and gene mutations in CCAs patients. (A) Somatic Cell Mutation Profiles in 70 CCAs Patients. (B) Gene Mutation Mapping in 70 Patients with CCAs. (C–E) Comparison of Mutated Genes in TCGA‐CCAs and CCAs1‐70.

This study included 70 CCA patients for WES at Peking Union Medical College Hospital. Patient ages ranged from 37 to 82 years (median age: 63), with clinical characteristics detailed in Table S1. For the entire sample, 80.09% (5432/6782) of mutations were SNVs and 9.10% (617/6782) were insertion‐deletions (Indels). The top three somatic mutation types were C > T (54.84%), C > A (12.13%), and T > C (9.7%) (Figure 1A). A median of 72 somatic mutations were observed in each patient (range: 7–802). Among all mutations, genes that were mutated in at least two patients were selected for further analysis. A total of 4668 mutations were obtained from 70 samples, of which 25.17% (1175/4668) were mutated in at least two patients. TP53 was the most frequently mutated gene (37/70, 53%), followed by MUC17 (17/70, 24%), FLG (14/70, 20%), MUC4 (13/70, 19%), FLG2 (12/70, 17%), MUC16 (11/70, 16%), and AHNAK (10/70, 14%) (Figure 1B). Comparative analysis with The Cancer Genome Atlas Program (TCGA)‐CCAs database revealed 1132 common genes (Figure 1C). In our study, six of the ten most commonly mutated genes exhibited higher frequencies compared to those in the TCGA database (Figure 1D). This discrepancy may be attributed to the limited number of patients or potential racial differences. Additionally, we identified 43 previously unreported mutated genes. Of these, 27 genes were found in at least two samples, and 13 genes were found in at least three samples (Figure 1E). Furthermore, an NTRK1 fusion was detected in one sample, with the results presented in Figure S1.

### 3.2 Transcriptome analysis of molecular mechanisms and pathway variations in distinct CCAs subgroups

**FIGURE 2:**
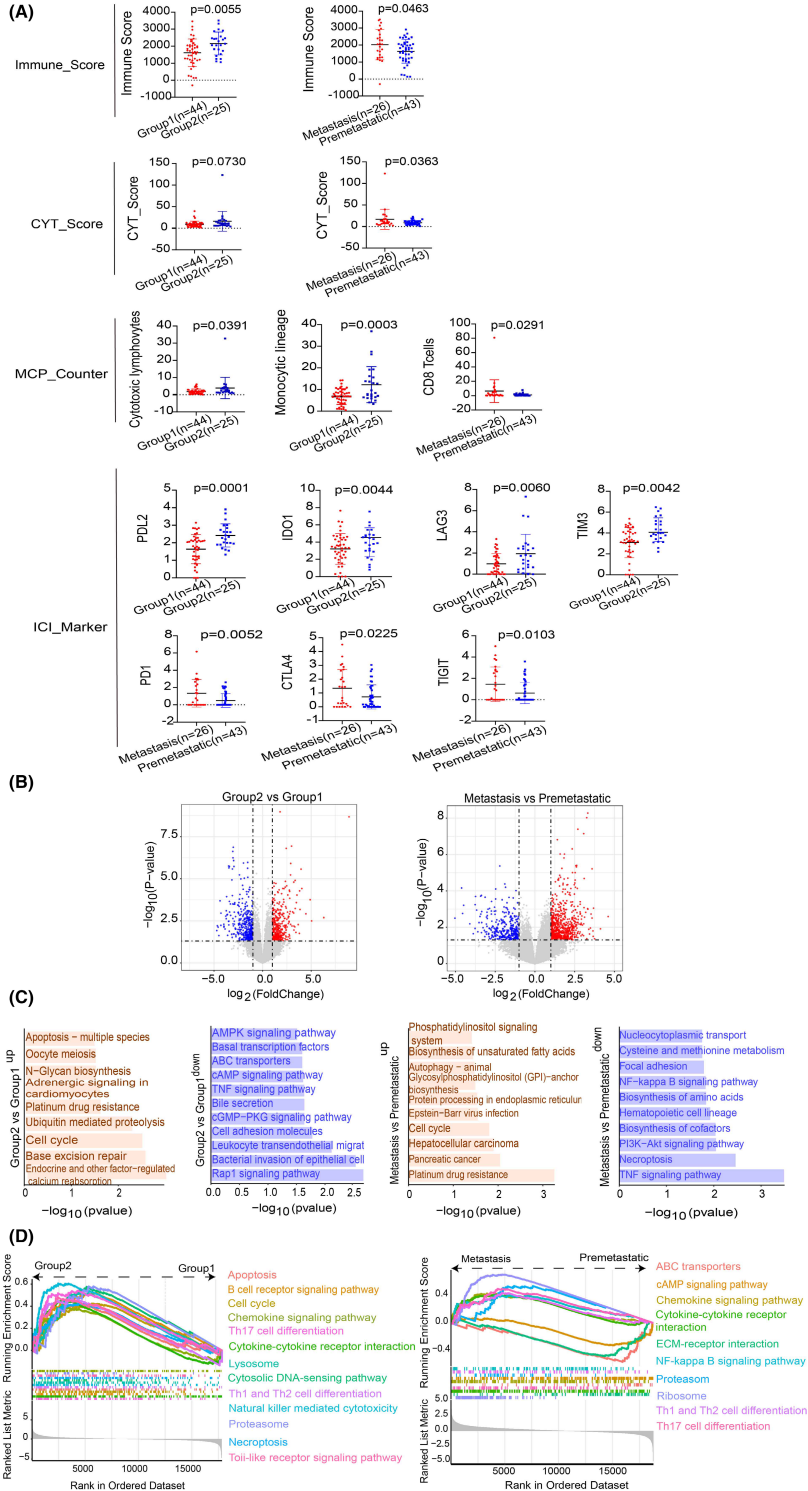
Disparities in molecular mechanisms and pathways at the transcriptome level across various subgroups. (A) Cellular immune infiltration and ICI marker gene expression. (B) Distinct gene expression profiles and pathway enrichment across different subgroups (gene expression volcano plot: Red dots indicate genes significantly upregulated in Group2 and the lymph node metastasis group. Blue dots represent genes significantly upregulated in Group1 and the group without lymph node metastasis. Gray dots indicate non‐significantly expressed genes). (C) Pathways significantly enriched in DEGs among different subgroups. (D) GSEA results between different subgroups.

We initially explored molecular mechanisms and pathways in CCAs patients, focusing on different degrees of differentiation and lymph node metastasis. Three immune infiltration scores (Immune, CYT, and MCP‐Counter) were calculated using RNA‐seq data. A comparison of immune infiltration between Group1 and Group2 revealed significantly lower Immune scores in Group1 (*p* = 0.0055), with no statistical significance in CYT scores (*p* > 0.05) (Figure 2A). In Group2, MCP‐Counter scores showed significantly higher levels of cytotoxic lymphocytes and monocytic lineage cell infiltration compared to Group1 (*p* = 0.0391 and *p* = 0.0003, respectively) (Figure 2A). Infiltration levels of T cells, CD8 T cells, NK cells, etc., did not significantly differ between the two groups (Table S2). Comparing the subgroups with and without lymph node metastasis, the immune and CYT scores were significantly higher in the metastasis group than in the group without lymph node metastasis (*p* = 0.0463 and *p* = 0.0363, respectively) (Figure 2A). In the MCP‐Counter score, the difference in the degree of cellular infiltration was not statistically significant (*p* > 0.05) except for CD8 T cells (*p* = 0.0291) (Figure 2A) (Table S2). Exploring potential immunosuppressive agents, gene expression levels of ICI were compared in different subgroups. Group1 exhibited significantly higher expression of PDL2, IDO1, LAG3, and TIM3 compared to Group2 (*p* = 0.0001, *p* = 0.0044, *p* = 0.0060, and *p* = 0.0042, respectively) (Figure 2A), while PD1, PDL1, and IDO2 did not show statistically significant differences (*p* > 0.05) (Table S4). In the lymph node metastasis analysis, PD1, CTLA4, and TIGIT had significantly higher expression in the metastatic group (*p* = 0.0052, *p* = 0.0225, and *p* = 0.0103) (Figure 2A), with no significant differences in PDL2, PDL1, and IDO1 expression (Table S3).

To discern DEGs and pivotal pathways, we conducted transcriptome comparisons among groups with varying degrees of differentiation and with or without lymph node metastasis. The results unveiled 876 significantly up‐regulated genes and 806 significantly down‐regulated genes in Group2 compared to Group1 (*p*‐value <0.05 and |log2(Fold Change)| ≥1). Likewise, 1636 genes were significantly up‐regulated, and 736 genes were significantly down‐regulated in the lymph node metastasis group compared to the non‐metastasis group (*p*‐value <0.05 and |log2(Fold Change)| ≥1) (Figure 2B). Subsequently, KEGG pathway enrichment analysis was employed to elucidate the functional roles of these DEGs and identify key pathways in our CCAs patients. A total of 47 pathways, including 16 up‐regulated genes and 31 down‐regulated genes, were enriched based on the *p*‐value <0.05 criterion in groups with different degrees of differentiation. Notably, the cell cycle and apoptosis‐multispecies pathways were significantly enriched in the Group2 group. In contrast, the Rap1 signaling pathway, TNF signaling pathway, and AMPK signaling pathway were significantly enriched in the Group1 group. For the group with or without lymph node metastasis, 33 pathways were enriched, encompassing 18 up‐regulated genes, and 15 down‐regulated genes. The group with lymph node metastasis exhibited significant enrichment in the platinum drug resistance and cell cycle pathways, while the group without lymph node metastasis showed enrichment in pathways like TNF signaling, PI3K‐Akt signaling, NF‐kappa B signaling, and nucleocytoplasmic transport. Figure 2C provides crucial information on the enriched pathways in both groups.

To delineate distinctions in functional signaling pathways, we employed GSEA to pinpoint the pathways enriched in different subgroups. In Group2, a total of 54 pathways exhibited significant enrichment, with Th17 cell differentiation, cytokine‐cytokine receptor interaction, B cell receptor signaling pathway, and cell cycle pathways displaying higher expression in Group2 (Figure 2D). Conversely, the group with lymph node metastasis demonstrated significant enrichment in 68 pathways. Noteworthy pathways with elevated expression in the lymph node metastasis group included ribosome, Th17 cell differentiation, proteasome, cytokine‐cytokine receptor interaction, and chemokine signaling pathway. Genes associated with ABC transporters, ECM‐receptor interaction, and the cAMP signaling pathway exhibited higher expression in the metastasis group without lymph node involvement (Figure 2D).

### 3.3 Different gene expression spectrum and pathway enrichment in the long‐ and short‐term groups

**FIGURE 3:**
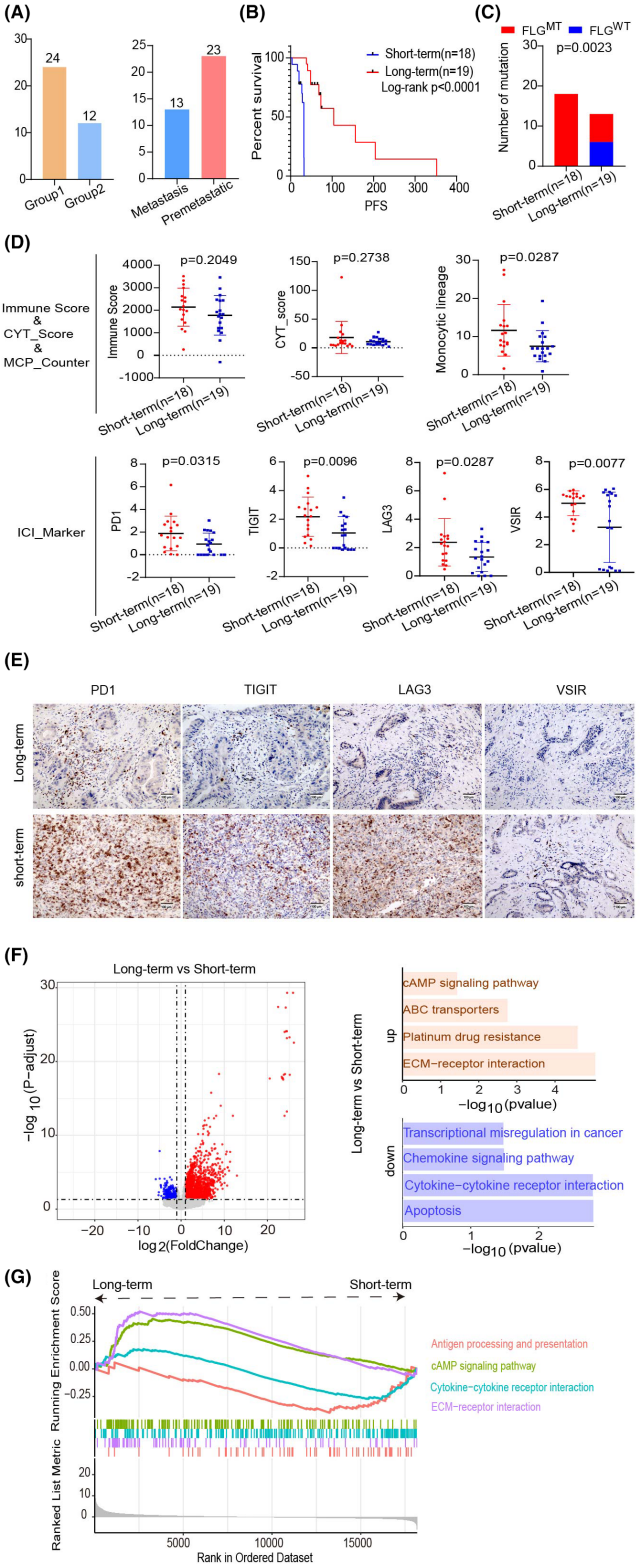
Illustrates the molecular mechanisms and pathway distinctions between the long‐term and short‐term groupings. (A) For samples with PFS data, the distribution of samples with different degrees of differentiation and lymph node metastasis status was analyzed. (B) Illustration of the relationship between PFS in long‐term and short‐term subgroups of CCA patients. (C) Comparison of the frequency of differential gene mutations between the two groups. (D) Evaluation of cellular immune infiltration and expression of ICI‐labeled genes (200×) between the two groups. Bar, 100 μm. (E) Immunohistochemical validation of ICI‐labeled gene expression between the two groups. (F) Differences in differential gene expression and pathway enrichment depicted through gene expression volcano plots (red dots indicate significantly higher gene expression in the long‐term group, while blue dots signify significantly higher gene expression in the short‐term group. Gray dots represent genes with no noticeable expression differences). (G) Presentation of GSEA results highlighting distinctions between the two groups.

To investigate the differences in gene mutations, gene expression, and immune infiltration among CCA patients with varying survival periods, researchers divided the samples into a short‐term survival group and a long‐term survival group. Among these samples, there were 24 well‐differentiated cases and 12 poorly differentiated cases; 13 cases with lymph node metastasis, and 23 cases without lymph node metastasis (Figure 3A). A significant difference in PFS survival was observed between the two groups (*p* < 0.001) (Figure 3B). The somatic mutation results indicate that TP53 had the highest mutation frequency at 57%, followed by AHNAK2, ARID1A, FLG, and MUC16, all at 16%. Additionally, KMT2C and other genes had a mutation frequency of 14%, with FLG appearing exclusively in the long‐term group (*p* = 0.0023) (Figure 3C). However, no significant differences (*p* > 0.05) were found for the other genes between the two groups (Table S4). There was no significant distinction (*p* > 0.05) in CYT and Immune scores between the two groups (Figure 3D). In the MCP‐Counter score results, Monocytic Lineage (*p* = 0.0287) showed a significant difference in cellular infiltration between the groups (Figure 3D), while the levels of cellular infiltration for other cells were not significantly different (*p* > 0.05) (Table S2). ICI marker results were not significant (*p* > 0.05) except for PD1, TIGIT, CTLA4, and VSIR, which showed significant differences between the groups (Figure 3D,E, Table S3).

Differential gene expression analysis identified 2645 up‐regulated and 499 down‐regulated genes in the long‐term group (*p*‐value <0.05 and |log2 (Fold Change) | ≥1). KEGG pathway enrichment analysis uncovered 47 significantly enriched pathways, Among these, 36 up‐regulated genes were associated with pathways such as the cAMP signaling pathway, ABC transporters, platinum drug resistance, and ECM‐receptor interaction. Conversely, 11 down‐regulated genes were linked to pathways such as transcriptional misregulation in cancer, chemokine signaling pathway, and cytokine‐cytokine receptor interaction (Figure 3F). Further characterization of functional signaling pathways through GSEA showed significant elevation of ECM‐receptor interaction and cAMP signaling pathway genes in the long‐term group, while antigen processing and presentation, and cytokine‐cytokine receptor interaction were notably elevated in the short‐term group (Figure 3G).

### 3.4 Correlation between varied differentiation degrees and lymph node metastasis with PFS in CCAs patients

**FIGURE 4:**
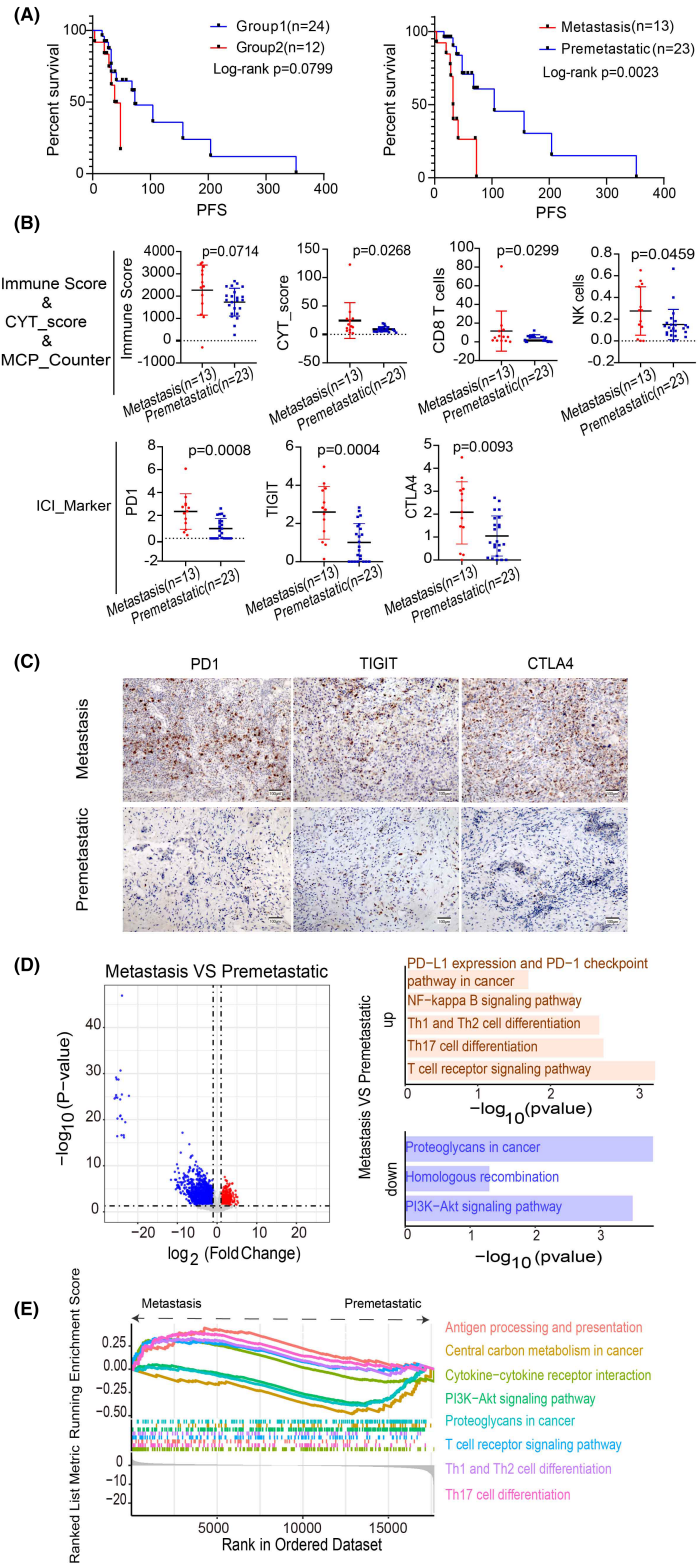
Depicts the association between differentiation levels, lymph node metastasis, and PFS in CCAs patients, emphasizing variations in molecular mechanisms and pathways. (A) Description of the relationship between PFS in different subgroups. (B) Assessment of cellular immune infiltration and expression of ICI marker genes between lymph node metastasis groups. (C) Immunohistochemical validation of ICI‐labeled gene expression (200×) between the two groups. Bar, 100 μm. (D) Differences in gene expression and pathway enrichment are depicted by gene expression volcano plots (red dots indicate significantly higher gene expression in the lymph node metastasis group and blue dots indicate significantly higher gene expression in the lymph node non‐metastasis group. Gray dots represent genes with no significant expression differences). (E) GSEA results highlighting distinctions between the two groups.

To further explore the influence of differentiation degree and lymph node metastasis, two risk factors, on PFS, we stratified PFS according to these risk factors. Notably, there was a significant difference in PFS in the lymph node metastasis group (*p* = 0.0023). However, differences in PFS among groups with varying degrees of differentiation did not achieve statistical significance, possibly due to the small sample size (Figure 4A). In the lymph node metastasis group, TP53 was the most frequently mutated gene at 57%, followed by AHNAK2, ARID1A, FLG, and MUC16 at 16%. Additionally, genes like KMT2C were mutated in 14% of cases, but none of these gene mutations reached statistical significance (*p* > 0.05) between the two groups (Table S4).

The CYT score in the lymph node metastasis group was significantly higher than that in the non‐metastasis group (*p* = 0.0268), while the Immune score did not show statistical significance (*p* > 0.05). MCP‐Counter scores revealed that CD8 T cells and NK cells were significantly more abundant in the metastasis group compared to the non‐metastasis group (*p* = 0.0299, *p* = 0.0459). Other cell infiltration levels did not exhibit significance (Table S2). ICI marker analysis indicated that the lymph node metastasis group exhibited non‐significant differences in immune checkpoint gene expression levels, except for PD1, TIGIT, and CTLA4, which were significantly higher than those in the non‐metastasis group (*p* = 0.0008, *p* = 0.0004, *p* = 0.0093) (Table S3). The significant differences in ICI markers were further confirmed through immunohistochemistry (Figure 4B,C).

We conducted a comparative analysis of differential gene expression between the two groups. The results identified 1049 significantly up‐regulated genes and 2439 significantly down‐regulated genes in the group with lymph node metastasis (*p*‐value <0.05 and |log2 (Fold Change) |≥1). Subsequently, KEGG pathway enrichment analysis was performed to elucidate the functional roles of these DEGs. The analysis revealed 60 significantly enriched pathways in the lymph node metastasis group. Of these, 29 up‐regulated genes were involved in pathways such as the T cell receptor signaling pathway, Th17 cell differentiation, Th1 and Th2 cell differentiation, NF‐kappa B signaling pathway, and PD‐L1 expression and PD‐1 checkpoint pathway in cancer. Additionally, 31 down‐regulated genes, were associated with pathways such as proteoglycans in cancer, PI3K‐Akt signaling pathway, and homologous recombination (Figure 4D). Functional signaling pathway disparities between the two groups were further characterized using GSEA. Genes associated with cytokine‐cytokine receptor interaction, Th17 cell differentiation, antigen processing and presentation, T cell receptor signaling pathway, and Th1 and Th2 cell differentiation were significantly upregulated in the lymph node metastasis group. In contrast, genes related to the PI3K‐Akt signaling pathway, central carbon metabolism in cancer, and proteoglycans in cancer were notably elevated in the lymph node non‐metastasis group (Figure 4E).

### 3.5 The relationship between TMB, ITH, and patient PFS

As reported in previous studies, TMB and ITH are recognized as factors associated with cancer prognosis.^16^, ^17^ Therefore, this study investigated the correlation between these two factors and patient prognosis. TMB values ranged from 0.20 to 13.21 (median = 1.09), while mutant‐allele tumor heterogeneity (MATH) values ranged from 0.0 to 89.66 (median = 36.66). TMB and MATH values were individually compared between the short‐term and long‐term subgroups, but no significant differences were observed (Figure S2A). Subsequently, all patients were regrouped based on the median values of TMB and ITH. The analysis did not reveal a significant correlation between TMB, ITH, and PFS in CCA patients (Figure S2B). We therefore hypothesized that high TMB and low ITH might not be associated with PFS in CCA patients not receiving immunotherapy.

## 4 DISCUSSION

This study deepens our understanding of how the two risk factors, differentiation levels, and lymph node metastasis, affect the survival of CCA patients. It uncovers associated molecular mechanisms and pathways, offering potential therapeutic strategies. The analysis involved gene mutations and immunity status in 70 Chinese CCA patients. Genes with the highest mutation frequencies exceeded those in the TCGA data. Additionally, 43 genes were exclusively mutated in our study, possibly due to ethnic differences or the limited patient cohort. FLG genes were exclusively detected in the long‐term PFS subgroup, suggesting a potential association with better prognosis. Although there is currently no direct evidence linking FLG genes to prognosis, this finding opens new avenues for research into the prognostic mechanisms of CCA. Furthermore, guidelines recommend considering targeted therapies with entrectinib and larotrectinib for CCA patients with NTRK1 gene fusions.

High TMB is often linked to poorer prognosis in many cancers.^16^, ^17^ In our study, similar to TMB, ITH also showed comparable results, indicating no association between PFS and TMB or ITH. The precise reason for this inconsistency remains unclear and may be influenced by factors like tumor histological type, treatment modalities, or sample size.

Cellular immune infiltration was higher in the low‐differentiation group (indicated by the Immune score) and in the group with lymph node metastasis (showing elevated Immune and CYT scores). Specifically, levels of cytotoxic lymphocytes and monocytic lineage cell infiltration were higher in the poorly differentiated group compared to the highly‐moderately differentiated group. CD8T cells and NK cells, along with the lymph node metastasis subgroup in the PFS survival analysis, were higher in the metastasis group than in the non‐metastasis group. Monocytic lineage cell infiltration was also higher than in the long‐term group, suggesting an enhanced autoimmune response during tumor development. Expression levels of ICI marker genes and immunohistochemistry results indicated that ICI effectiveness, such as PDL2, IDO1, and LAG3, was higher in the highly‐moderately differentiated group than in the poorly differentiated group. Similarly, ICI effectiveness, including PD1, CTLA4, and TIGIT, was higher in the group with lymph node metastasis than in the non‐metastasis group. The effects of ICIs like PD1, TIGIT, and LAG3 in the PFS short‐term group were higher than those in the long‐term group. Given the low TMB results for all patients (ranging from 0.20 to 13.21), indicating unfavorable outcomes for immunotherapy, it is hypothesized that immunotherapy alone, such as checkpoint inhibitors, is less likely to achieve a high remission rate in patients with CCAs.

In KEGG analysis, the low differentiation group exhibited notable enrichment in the cell cycle and apoptosis pathways, indicating aggressive CCAs with a promotion of tumor growth and progression.^18^, ^19^, ^20^, ^21^ The lymph node metastasis group displayed enrichment in platinum drug resistance, cell cycle, and PD‐L1 expression/PD‐1 checkpoint pathway in cancer pathways. This suggests potential resistance to platinum‐based chemotherapy, requiring alternative therapeutic strategies.^22^, ^23^ The group without lymph node metastasis showed enrichment in the TNF signaling pathway, PI3K‐Akt signaling pathway, and proteoglycans in cancer pathways, possibly associated with a better prognosis.^24^, ^25^, ^26^ The significant enrichment of the cAMP signaling pathway in the long survival group provides insights into factors contributing to the improved prognosis. Conversely, the significant enrichment of transcriptional misregulation in cancer in the short survival group suggests that these pathways could be potential targets for therapeutic intervention. Targeting these pathways may hold promise for the development of more effective therapies, ultimately enhancing patient outcomes.

In GSEA, genes linked to Th17 cell differentiation, cytokine‐cytokine receptor interaction, B cell receptor signaling, and the cell cycle were upregulated in poorly differentiated tumors. This suggests a more active immune response and increased tumor cell proliferation, indicating heightened invasiveness.^27^, ^28^, ^29^ This gene expression profile indicates that poorly differentiated tumors typically have a worse prognosis, suggesting these genes could serve as biomarkers for identifying patients at higher risk of disease progression. Elevated expression in pathways like Th17 cell differentiation, proteasome, and cytokine‐cytokine receptor interaction suggests strong tumor invasiveness, explaining the occurrence of lymph node metastasis.^30^ Moreover, cAMP signaling pathway genes were notably increased in the long‐term group, suggesting that boosting this pathway can hinder cellular proliferation, potentially improving prognosis.

## 5 CONCLUSIONS

In summary, this study primarily investigates the mutation spectrum, immune infiltration, and their impact on clinical prognosis in CCA, with particular emphasis on the molecular mechanisms of two key risk factors, notably lymph node metastasis. The study findings suggest that relying solely on immunotherapy may not lead to significant relief for CCA patients. For poorly differentiated CCA, intervention in tumor metabolism and inhibition of tumor growth may be attempted through targeting the TNF signaling pathway and modulating the AMPK signaling pathway. Additionally, patients with lymph node metastasis, a significant risk factor impacting PFS, may benefit from combination therapy using targeted drugs to overcome platinum‐based chemotherapy resistance. These findings provide important guidance for current research and treatment of CCA, with the potential to improve patient prognosis.

## AUTHOR CONTRIBUTIONS

**Baoluhe Zhang:** Conceptualization (equal); formal analysis (equal); methodology (equal); writing – original draft (equal). **Bao Jin:** Conceptualization (equal); formal analysis (equal); investigation (equal); software (equal); visualization (equal). **Xiang'an Wu:** Software (equal). **Jiali Xing:** Validation (equal). **Xiao Liu:** Data curation (equal). **Xueshuai Wan:** Data curation (equal). **Haifeng Xu:** Writing – review and editing (equal). **Yiyao Xu:** Resources (equal). **Yilei Mao:** Visualization (equal); writing – review and editing (equal). **Qian Chen:** Formal analysis (equal). **Yating Bai:** Formal analysis (equal). **Mei Guan:** Software (equal). **Shunda Du:** Funding acquisition (equal); investigation (equal); project administration (equal); supervision (equal); writing – review and editing (equal).

## FUNDING INFORMATION

This work was supported by grants from the National Natural Science Foundation of China (Grant Number: 81972698), National High Level Hospital Clinical Research Funding (No. 2022‐PUMCH‐C‐047), CAMS Innovation Fund for Medical Sciences (CIFMS) (No. 2021‐I2M‐1‐014) and the Fundamental Research Funds for the Central Universities (No: 3332023009).

## CONFLICT OF INTEREST STATEMENT

The authors declare that the research was conducted in the absence of any commercial or financial relationships that could be construed as a potential conflict of interest.

## ETHICS STATEMENT

The study, approved Ethics Review Committee of Peking Union Medical College Hospital (I‐23PJ1691).

## Supporting information


Data S1.

## Data Availability

The data that support the findings of this study are available from the corresponding author upon reasonable request.
