# Supplementary material for: Investigation of transcriptional and immunological disparities among patient groups with varied prognostic risk factors in cholangiocarcinoma
Source: Cancer Med. 2024 Aug 29;13(16):e70135. doi: 10.1002/cam4.70135 (PMC11358702; doi:10.1002/cam4.70135)
Supplement: Supplementary file 1 — Data S1. [file CAM4-13-e70135-s001.docx]

Supplementary Table 1：Clinical features of the studied 70 CCAs patients

| **SampleID** | **Gender** | **Age** | **Tumor_differentiation** | **Lymph Premetastaticde/ Metastases** | **Long-term/short-term** | **Basic Liver Disease** | **Anatomical location of the tumor** | **Radiotherapy Chemotherapy** | **Immunotherapy treatment** |
| --- | --- | --- | --- | --- | --- | --- | --- | --- | --- |
| Pt01 | M | 77 | Group2 | Premetastatic | NA | No | Lower Part of the Common Bile Duct | No | No |
| Pt02 | M | 47 | Group1 | Premetastatic | NA | No | Porta hepatis | No | No |
| Pt03 | F | 55 | Group2 | Metastasis | NA | No | Lower Part of the Common Bile Duct | No | No |
| Pt04 | M | 64 | Group1 | Metastasis | NA | Yes(Hepatitis B virus) | Upper part of the common bile duct | No | No |
| Pt05 | M | 61 | Group2 | Premetastatic | NA | No | Upper part of the common bile duct | No | No |
| Pt06 | M | 59 | Group1 | Premetastatic | NA | No | Upper part of the common bile duct | No | No |
| Pt07 | M | 63 | Group2 | Premetastatic | Long-term | No | Upper part of the common bile duct | No | No |
| Pt08 | F | 63 | Group2 | Metastasis | NA | No | Upper part of the common bile duct | No | No |
| Pt09 | M | 60 | Group2 | Premetastatic | NA | No | Upper part of the common bile duct | No | No |
| Pt10 | M | 64 | Group2 | Metastasis | NA | No | Upper part of the common bile duct | No | No |
| Pt11 | F | 57 | Group2 | Metastasis | NA | No | Lower Part of the Common Bile Duct | No | No |
| Pt12 | F | 68 | Group2 | Premetastatic | NA | No | Lower Part of the Common Bile Duct | No | No |
| Pt13 | M | 66 | Group2 | Premetastatic | NA | No | Upper part of the common bile duct | No | No |
| Pt14 | F | 57 | Group1 | Metastasis | NA | No | Upper part of the common bile duct | No | No |
| Pt15 | M | 54 | Group1 | Metastasis | NA | No | Upper part of the common bile duct | No | No |
| Pt16 | M | 64 | Group1 | Premetastatic | Long-term | No | Upper part of the common bile duct | No | No |
| Pt17 | M | 59 | Group1 | Premetastatic | NA | No | Upper part of the common bile duct | No | No |
| Pt18 | F | 76 | Group1 | Metastasis | NA | No | Upper part of the common bile duct | No | No |
| Pt19 | M | 63 | Group2 | Premetastatic | NA | No | Lower Part of the Common Bile Duct | No | No |
| Pt20 | F | 44 | Group2 | Premetastatic | NA | No | Upper part of the common bile duct | No | No |
| Pt21 | F | 63 | Group1 | Premetastatic | NA | No | Lower Part of the Common Bile Duct | No | No |
| Pt22 | M | 68 | Group2 | Premetastatic | Long-term | No | Upper part of the common bile duct | No | No |
| Pt23 | M | 72 | Group2 | Metastasis | NA | No | Lower Part of the Common Bile Duct | No | No |
| Pt24 | M | 75 | Group1 | Premetastatic | Long-term | No | Lower Part of the Common Bile Duct | No | No |
| Pt25 | F | 50 | Group1 | Premetastatic | NA | No | Lower Part of the Common Bile Duct | No | No |
| Pt26 | M | 47 | Group1 | Premetastatic | Long-term | No | Upper part of the common bile duct | No | No |
| Pt27 | M | 50 | Group1 | Premetastatic | NA | No | Lower Part of the Common Bile Duct | No | No |
| Pt28 | M | 48 | Group1 | Premetastatic | NA | No | Lower Part of the Common Bile Duct | No | No |
| Pt29 | M | 59 | Group1 | Premetastatic | NA | No | Lower Part of the Common Bile Duct | No | No |
| Pt30 | F | 64 | Group1 | Premetastatic | Long-term | No | Lower Part of the Common Bile Duct | No | No |
| Pt31 | F | 37 | Group1 | Metastasis | NA | No | Lower Part of the Common Bile Duct | No | No |
| Pt32 | F | 62 | Group1 | Metastasis | NA | No | Lower Part of the Common Bile Duct | No | No |
| Pt33 | M | 56 | Group1 | Premetastatic | NA | No | Upper part of the common bile duct | No | No |
| Pt34 | F | 67 | Group1 | Premetastatic | NA | No | Lower Part of the Common Bile Duct | No | No |
| Pt35 | F | 60 | Group1 | Premetastatic | NA | No | Lower Part of the Common Bile Duct | No | No |
| Pt36 | F | 58 | Group1 | Premetastatic | NA | No | Upper part of the common bile duct | No | No |
| Pt37 | F | 63 | Group1 | Premetastatic | Long-term | No | Upper part of the common bile duct | No | No |
| Pt38 | F | 54 | Group1 | Metastasis | Short-term | No | Right hemiliver | No | No |
| Pt39 | M | 61 | Group1 | Metastasis | Long-term | No | Upper part of the common bile duct | No | No |
| Pt40 | M | 67 | Group1 | Premetastatic | Short-term | No | Upper part of the common bile duct | No | No |
| Pt41 | M | 66 | Group1 | Premetastatic | Long-term | No | Upper part of the common bile duct | No | No |
| Pt42 | M | 73 | Group2 | Metastasis | NA | No | Lower Part of the Common Bile Duct | No | No |
| Pt43 | F | 61 | Group1 | Premetastatic | Long-term | No | Lower Part of the Common Bile Duct | No | No |
| Pt44 | F | 58 | Group1 | Metastasis | Long-term | No | Lower Part of the Common Bile Duct | No | No |
| Pt45 | M | 77 | Group2 | Premetastatic | Long-term | No | Lower Part of the Common Bile Duct | No | No |
| Pt46 | M | 69 | NA | Premetastatic | Long-term | No | Lower Part of the Common Bile Duct | No | No |
| Pt47 | M | 67 | Group2 | NA | Short-term | No | Upper part of the common bile duct | No | No |
| Pt48 | M | 64 | Group1 | Premetastatic | Long-term | No | Lower Part of the Common Bile Duct | No | No |
| Pt49 | M | 64 | Group2 | Premetastatic | Long-term | No | Upper part of the common bile duct | No | No |
| Pt50 | M | 49 | Group1 | Metastasis | Short-term | No | Lower Part of the Common Bile Duct | No | No |
| Pt51 | M | 67 | Group1 | Metastasis | Short-term | Yes(Hepatitis B virus) | Left half of the liver | No | No |
| Pt52 | F | 70 | Group1 | Premetastatic | Long-term | No | Upper part of the common bile duct | No | No |
| Pt53 | F | 71 | Group2 | Premetastatic | Long-term | No | Upper part of the common bile duct | No | No |
| Pt54 | M | 62 | Group1 | Metastasis | Long-term | No | Porta hepatis | No | No |
| Pt55 | M | 54 | Group1 | Premetastatic | Short-term | No | Lower Part of the Common Bile Duct | No | No |
| Pt56 | M | 64 | Group2 | Metastasis | Short-term | No | Lower Part of the Common Bile Duct | No | No |
| Pt57 | F | 58 | Group1 | Metastasis | Long-term | No | Lower Part of the Common Bile Duct | No | No |
| Pt58 | F | 53 | Group2 | Metastasis | Short-term | No | Upper part of the common bile duct | No | No |
| Pt59 | M | 69 | Group1 | Metastasis | Short-term | No | Porta hepatis | No | No |
| Pt60 | M | 69 | Group2 | Premetastatic | Short-term | No | Left half of the liver | No | No |
| Pt61 | M | 72 | Group2 | Metastasis | Short-term | No | Lower Part of the Common Bile Duct | No | No |
| Pt62 | M | 63 | Group2 | Premetastatic | Short-term | No | Right hemiliver | No | No |
| Pt63 | M | 71 | Group1 | Metastasis | Short-term | No | Upper part of the common bile duct | No | No |
| Pt64 | F | 70 | Group1 | Premetastatic | NA | No | Porta hepatis | No | No |
| Pt65 | M | 82 | Group1 | Premetastatic | Short-term | No | Right hemiliver | No | No |
| Pt66 | M | 51 | Group1 | Metastasis | NA | No | Left half of the liver | No | No |
| Pt67 | M | 50 | Group1 | Premetastatic | Short-term | No | Left half of the liver | No | No |
| Pt68 | F | 74 | Group1 | Premetastatic | Short-term | No | Left half of the liver | No | No |
| Pt69 | M | 53 | Group2 | Metastasis | Short-term | No | Right hemiliver | No | No |
| Pt70 | F | 69 | Group1 | Premetastatic | Short-term | No | Lower Part of the Common Bile Duct | No | No |

Footnotes: Group1, Highly differentiated and moderately differentiated tumors; Group2, Hypodifferentiation (Including hypo- and meso-hypodifferentiation); Long-term （PFS≥ 38.1 weeks）,short-term (PFS<38.1 weeks).


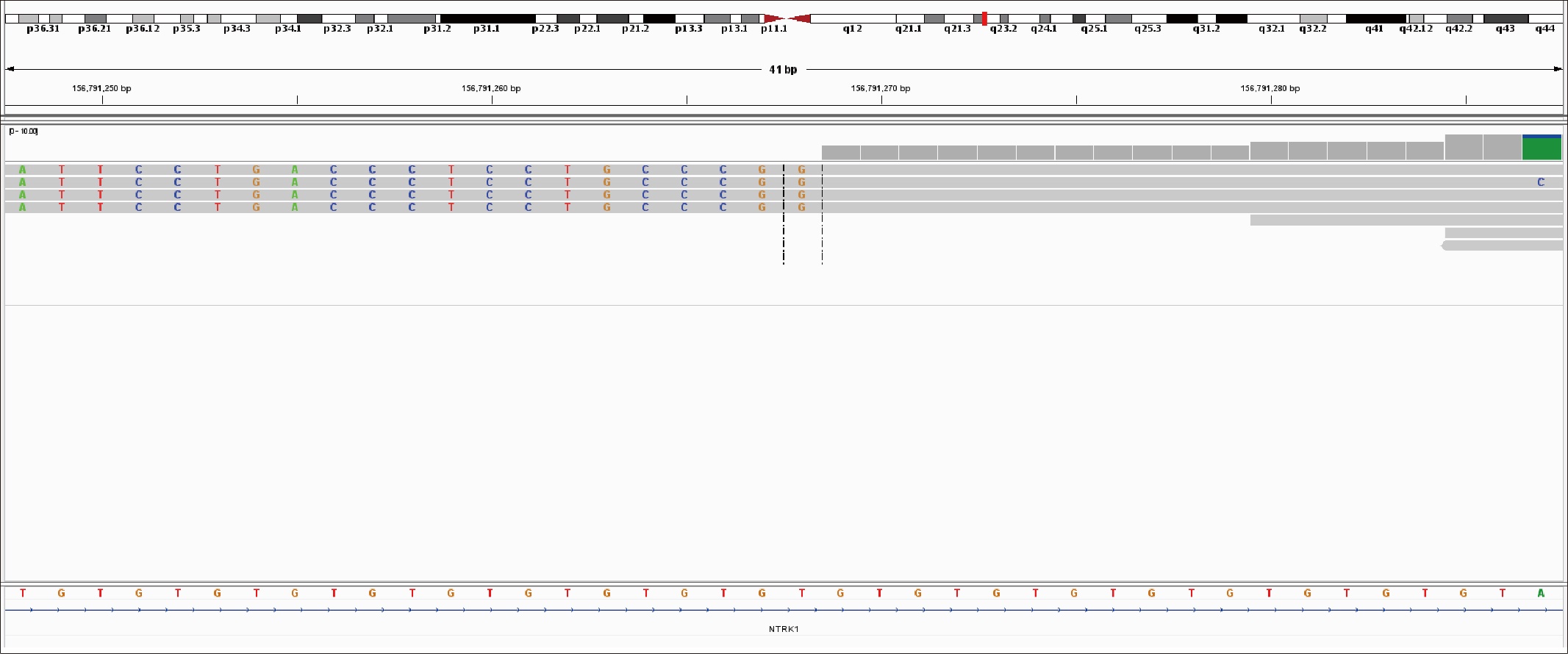


Supplementary Figure 1 NTRK1- ETV4 gene fusion visualization map

Supplementary Table2: Comparison of results of immune cell infiltration in different subgroups

| Group | | | | | | MCP_counter | | | | | |
| --- | --- | --- | --- | --- | --- | --- | --- | --- | --- | --- | --- |
| / | T cells | CD8 T cells | Cytotoxic lymphocytes | NK cells | B lineage | | Monocytic lineage | Myeloid dendritic cells | Neutrophils | Endothelial  cells | Fibroblasts |
| Group1 vs Group2(all) P value | 0.14 | 0.10 | 0.04 | 0.06 | 0.13 | | <0.01 | 0.08 | 0.48 | 0.87 | 0.06 |
| Metastasis vs Premetastatic(all) P value | 0.16 | 0.03 | 0.06 | 0.26 | 0.09 | | 0.24 | 0.07 | 0.24 | 0.50 | 0.17 |
| Metastasis vs Premetastatic(PFS) P value | 0.07 | 0.03 | 0.07 | 0.05 | 0.05 | | 0.84 | 0.11 | 0.16 | 0.26 | 0.07 |
| Short_term VS Long_term(PFS) P value | 0.83 | 0.18 | 0.32 | 0.81 | 0.41 | | 0.03 | 0.95 | 0.88 | 0.88 | 0.07 |

Supplementary Table3: Comparison of results of ICI_Marker in different subgroups

| Group | | | ICI_Marker | | | | | | | | | | |
| --- | --- | --- | --- | --- | --- | --- | --- | --- | --- | --- | --- | --- | --- |
| / | PD1 | PDL2 | | PDL1 | IDO1 | IDO2 | CTLA4 | ADORA2A | TIGIT | LAG3 | VTCN1 | TIM3 | VSIR |
| Group1 vs Group2(all)  P value | 0.43 | <0.01 | | 0.09 | <0.01 | 0.05 | 0.21 | 0.17 | 0.80 | <0.01 | 0.07 | 0.04 | 0.94 |
| Metastasis vs Premetastatic(all)  P value | <0.01 | 0.84 | | 0.26 | 0.38 | 0.86 | 0.02 | 0.36 | 0.01 | 0.09 | 0.54 | 0.84 | 0.16 |
| Metastasis vs Premetastatic(PFS)  P value | <0.01 | 0.46 | | 0.07 | 0.87 | 0.37 | <0.01 | 0.35 | <0.01 | 0.10 | 0.36 | 0.36 | 0.06 |
| Short_term VS Long_term(PFS)  P value | 0.03 | 0.66 | | 0.07 | 0.59 | 0.37 | 0.14 | 0.32 | 0.01 | 0.03 | 0.38 | 0.13 | 0.01 |

Supplementary Table4: Comparison of mutation frequencies in different subgroups (showing top 10)

| Somatic Mutation | TP53 | FLG | MUC16 | ARID1A | AHNAK2 | KMT2C | ZNF729 | RBM10 | KRAS | ZNF469 |
| --- | --- | --- | --- | --- | --- | --- | --- | --- | --- | --- |
| Metastasis vs  Premetastatic (PFS) P value | 0.30 | 0.07 | 0.39 | 0.65 | 0.39 | >0.99 | 0.63 | 0.33 | 0.33 | >0.99 |
| Short-term vs Long-term (PFS) P value | 0.70 | 0.01 | 0.68 | 0.36 | 0.68 | >0.99 | 0.13 | >0.99 | >0.99 | 0.37 |


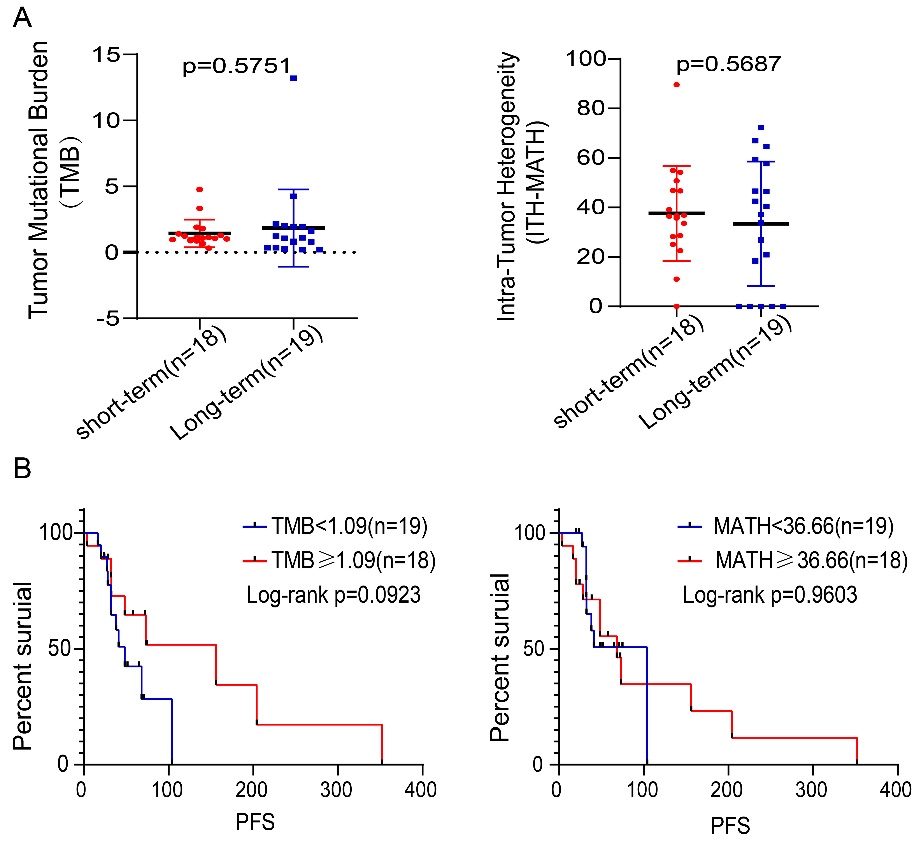


Supplementary Figure 2 Depicts the relationship between TMB, MTAH and PFS

A. TMB and MATH between short-term and long-term groups. B. Relationship between TMB, MATH, and the prognosis of CCAs patie.
